# Association between Serum Lactate Dehydrogenase Level and 30-day Mortality in Patients with Intracranial Hemorrhage with Acute Leukemia in the Induction Phase: A Cohort Study

**DOI:** 10.1055/s-0044-1786005

**Published:** 2024-04-10

**Authors:** Jia-Yuan Zhang, Zhang-Song Yan, Xiu-Juan Sun, Yong-Ze Liu, Yan-Ke Yin, Ming-Huan Su, Qiu-Ling Li, Ying-Chang Mi, Da-Peng Li

**Affiliations:** 1 Department of Emergency, State Key Laboratory of Experimental Hematology, National Clinical Research Center for Blood Diseases, Haihe Laboratory of Cell Ecosystem, Institute of Hematology and Blood Diseases Hospital, Chinese Academy of Medical Sciences and Peking Union Medical College, Tianjin, China

**Keywords:** lactate dehydrogenase, intracranial hemorrhage, acute leukemia

## Abstract

**Objectives** This study aimed to identify the association between lactate dehydrogenase (LDH) levels and 30-day mortality in patients with intracranial hemorrhage (ICH) with acute leukemia during the induction phase.

**Methods** This cohort study included patients with acute leukemia with ICH during induction. We evaluated serum LDH levels upon admission. Multivariable Cox regression analyzed the LDH 30-day mortality association. Interaction and stratified analyses based on factors like age, sex, albumin, white blood cell count, hemoglobin level, and platelet count were conducted.

**Results** We selected 91 patients diagnosed with acute leukemia and ICH. The overall 30-day mortality rate was 61.5%, with 56 of the 91 patients succumbing. Among those with LDH levels ≥ 570 U/L, the mortality rate was 74.4% (32 out of 43), which was higher than the 50% mortality rate of the LDH < 570 U/L group (24 out of 48) ( *p* = 0.017). In our multivariate regression models, the hazard ratios and their corresponding 95% confidence intervals for Log2 and twice the upper limit of normal LDH were 1.27 (1.01, 1.58) and 2.2 (1.05, 4.58), respectively. Interaction analysis revealed no significant interactive effect on the relationship between LDH levels and 30-day mortality.

**Conclusions** Serum LDH level was associated with 30-day mortality, especially in patients with LDH ≥ 570 U/L.

## Introduction

In patients with acute leukemia undergoing the induction phase, intracranial hemorrhage (ICH) is a severe complication. The reported 30-day mortality rate varies between 33 and 67%, 1 2 and survivors may experience significant long-term health issues. Early mortality refers to any cause of death within 30 days of diagnosis and poses a critical clinical challenge that hematologists aim to address, 3 4 as it represents an unresolved issue. Previous studies have identified factors such as advanced age, multisite hemorrhage (MSH), and altered consciousness as contributors to an increased risk of 30-day mortality. 5

Serum lactate dehydrogenase (LDH) levels are commonly used to evaluate disease severity and prognosis of various hematological cancers, such as acute myeloid leukemia (AML), myelodysplastic syndromes (MDS), and multiple myeloma (MM). 6 7 However, the relationship between LDH levels and 30-day mortality remains unclear, particularly in patients with ICH with acute leukemia during the induction phase. Additionally, we found no similar studies in the Chinese population. Early mortality is a critical concern in clinical practice; therefore, it is essential to investigate the connection between LDH levels and 30-day mortality to assist clinicians in making informed decisions. Hence, this study aimed to explore the link between LDH levels and 30-day mortality in patients with ICH with acute leukemia during the induction phase.

## Methods

### Patient Population

We conducted a retrospective analysis of 238 patients with hematological diseases and confirmed ICH through imaging. These patients were treated at the Institute of Hematology and Blood Diseases Hospital in Tianjin, China between January 2015 and April 2020. The study received approval from the Institutional Review Board of the Institute of Hematology and Blood Diseases Hospital.

The diseases in our study encompassed both acute and nonacute leukemia. Acute leukemia subtypes included acute lymphoblastic leukemia, AML, and acute mixed phenotype leukemia. Nonacute leukemia encompassed a range of conditions, such as MDS, atypical chronic myeloid leukemia (aCML), chronic myeloid leukemia (CML), chronic myelomonocytic leukemia (CMML), aplastic anemia (AA), immune thrombocytopenia, hemophilia, acquired hemophilia, vitamin-dependent coagulation factor deficiency, hereditary coagulation factor VII deficiency, non-Hodgkin's lymphoma (NHL), MM, pure red blood cell AA (PRCA), Evans syndrome, Fanconi anemia, connective tissue diseases associated with abnormal blood cell counts, and hypersplenism.

**Fig. 1:**
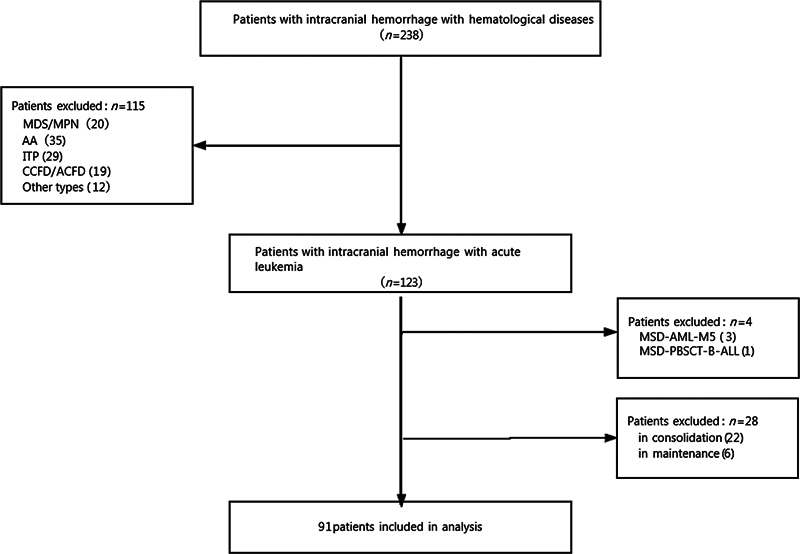
Patient selection flowchart. AA, aplastic anemia; ALL, acute lymphoblastic leukemia; allo-HSCT, allogeneic hematopoietic stem cell transplantation; AML, acute myeloid leukemia; CCFD/ACFD, congenital/acquired coagulation factor deficiency; ITP, immune thrombocytopenia; MDS/MPN, myelodysplastic/myeloproliferative neoplasms; MSD-PBSCT, matched sibling donor peripheral blood stem cell transplantation.

Patients with nonacute leukemia were categorized into three groups: (1) MDS/myeloproliferative neoplasms group, comprising patients with MDS, aCML, CML, and CMML; (2) congenital/acquired coagulation factor deficiency group, which included individuals with hemophilia, acquired hemophilia, vitamin-dependent coagulation factor deficiency, and hereditary coagulation factor VII deficiency; and (3) other group, which included patients with NHL, MM, PRCA, Evans syndrome, Fanconi anemia, connective tissue diseases with abnormal blood cell counts, and hypersplenism. We excluded 115 patients with nonacute leukemia, four with acute leukemia who underwent allogeneic hematopoietic stem cell transplantation, and 28 with acute leukemia in the consolidation or maintenance phase ( Fig. 1 ).

We conducted cranial computed tomography scans for all patients to assess the location and characteristics of cranial hemorrhages, as well as to document any accompanying signs, symptoms, or coinfections. However, determining the exact extent of the hemorrhage was challenging because of the diffuse and multifocal nature of most cases. ICHs were categorized into several types, including parenchymal hemorrhage (PCH), subarachnoid hemorrhage (SAH), subdural hemorrhage (SH), epidural hemorrhage (EH), and MSH. PCH referred to a single-site hemorrhage within the brain parenchyma. MSH included hemorrhages in multiple areas of the brain parenchyma, along with hemorrhages in other parts of the brain parenchyma. Other hemorrhage sites included SAH, SH, and EH. We also documented various signs and symptoms experienced by the patients, such as headaches, disturbances of consciousness, mental disorders, signs of meningeal irritation, hemiplegia, epilepsy, aphasia, and visual disturbances.

### Data Collection and Measurements

We gathered data, including survival status, from electronic medical records and follow-up phone calls. The baseline characteristics included age, sex, hypertension, diabetes mellitus, intracranial hemangioma, cranial trauma, primary disease, disease stage, and coinfections. Laboratory tests included white blood cell (WBC) count, hemoglobin (HGB) levels, platelet (PLT) counts, international normalized ratio (INR), partial thromboplastin time (APTT), fibrinogen (FIB) levels, D-Dimer (DD), serum urea, creatinine (Crea) levels, LDH, and peripheral blood blast counts. All data were collected within 3 days of the diagnosis of ICH in patients with acute leukemia.

We measured serum LDH levels using a biochemical analyzer equipped with an LDH test reagent (AU5800, Beckman Coulter, Inc.). The normal reference range for LDH was 80 to 285 U/L. Following the national standard, we divided LDH levels into two groups based on the upper limit of normal: <570 and ≥570 U/L. 8

### Intracranial Hemorrhage Management and Prognosis

In the medical management of ICH in individuals with acute leukemia, various treatment approaches are considered. These approaches can be broadly categorized into conservative and surgical interventions. Conservative treatment strategies primarily focus on supportive measures to address the bleeding and stabilize the patient. This includes therapies such as PLT transfusions, plasma transfusions, and FIB support to manage coagulation abnormalities. Hemostasis techniques and dehydration may also be employed to reduce intracranial pressure. Additionally, controlling the underlying leukemia and addressing any comorbidities are essential aspects of conservative management. Surgical interventions, on the other hand, are determined on a case-by-case basis by neurosurgeons following a thorough consultation and evaluation of the patient's condition. These interventions are tailored to the specific needs of the patient. The outcomes of these treatments are assessed by recording mortality events and their corresponding timeframes, typically within a 30-day period after ICH diagnosis.

### Statistical Analysis

We used multivariable Cox regression analyses to evaluate the independent association between serum LDH levels and 30-day mortality. Covariates-adjusted models were constructed using an extended Cox model approach. Subgroup analyses were conducted, stratified by relevant effect covariates.

A descriptive analysis was conducted for all participants. Categorical variables were presented as percentages (%), whereas continuous data were summarized as mean and standard deviation for normally distributed variables and as median and interquartile range (IQR) for skewed distributions. Variable comparisons were made using the chi-square test for categorical variables, one-way analysis of variance for normally distributed data, and the Kruskal–Wallis test for skewed data.

All statistical analyses were carried out using the R statistical software package, version 3.3.2 ( http://www.R-project.org , The R Foundation) and free statistics software version 1.4. Statistical significance was set at a two-tailed *p* -value of <0.05.

## Results

Table 1 summarizes the baseline characteristics of the study participants based on the LDH categories. Of the 238 patients, 91 met the inclusion criteria ( Fig. 1 ). The median LDH level was 531.2 U/L, with a range of 94.1 to 6987.3 U/L and an IQR of 270.4 to 1,521 U/L. The median age of the patients was 37.0 years, ranging between 2 to 76 years. Among the participants, 54 (59.3%) were men and 37 (40.7%) were women. Of the 91 patients, 35 had acute promyelocytic leukemia (APL), and the remaining 56 had other types of acute leukemia. Regarding the bleeding sites, 56 patients presented with MSH, 30 with PCH, and 5 with SAH, SH, or EH.

**Table 1:** Baseline characteristics of the study participants

Variables	Total ( *n* = 91)	LDH < 570 U/L ( *n* = 48)	LDH ≥ 570 U/L ( *n* = 43)	*p* -Value
Sex, *n* (%)				0.019
Male	54 (59.3)	23 (47.9)	31 (72.1)	
Female	37 (40.7)	25 (52.1)	12 (27.9)	
Age, median (IQR)	37.0 (17.5, 52.5)	43.0 (32.5, 55.8)	28.0 (15.0, 44.5)	0.003
Primary disease, *n* (%)				0.273
Non-APL	56 (61.5)	27 (56.2)	29 (67.4)	
APL	35 (38.5)	21 (43.8)	14 (32.6)	
Hypertension, *n* (%)				0.013
No	78 (85.7)	37 (77.1)	41 (95.3)	
Yes	13 (14.3)	11 (22.9)	2 (4.7)	
Diabetes, *n* (%)				0.119
No	87 (95.6)	44 (91.7)	43 (100)	
Yes	4 (4.4)	4 (8.3)	0 (0)	
Trauma history, *n* (%)				1
No	90 (98.9)	47 (97.9)	43 (100)	
Yes	1 (1.1)	1 (2.1)	0 (0)	
Bleeding site, *n* (%)				0.06
PCH	30 (33.0)	17 (35.4)	13 (30.2)	
SAH or SH or EH	5 (5.5)	5 (10.4)	0 (0)	
MSH	56 (61.5)	26 (54.2)	30 (69.8)	
Headache, *n* (%)				0.622
No	32 (35.2)	18 (37.5)	14 (32.6)	
Yes	59 (64.8)	30 (62.5)	29 (67.4)	
Disturbance of consciousness, *n* (%)				0.79
No	73 (80.2)	38 (79.2)	35 (81.4)	
Yes	18 (19.8)	10 (20.8)	8 (18.6)	
Hemiplegia, *n* (%)				0.539
No	58 (63.7)	32 (66.7)	26 (60.5)	
Yes	33 (36.3)	16 (33.3)	17 (39.5)	
Coinfection, *n* (%)				0.134
Yes	25 (27.5)	10 (20.8)	15 (34.9)	
No	66 (72.5)	38 (79.2)	28 (65.1)	
WBC, median (IQR)	37.0 (2.0, 203.5)	3.5 (0.7, 32.0)	201.9 (49.0, 385.0)	<0.001
Peripheral blast, median (IQR)	76.0 (6.0, 90.0)	35.0 (0.0, 72.8)	90.0 (84.0, 92.5)	<0.001
HGB, mean ± SD	78.5 ± 22.5	72.6 ± 18.2	85.1 ± 25.1	0.007
PLT, median (IQR)	21.0 (10.0, 34.0)	15.0 (5.0, 30.0)	27.0 (15.0, 36.0)	0.006
INR, mean ± SD	1.3 ± 0.3	1.2 ± 0.2	1.4 ± 0.3	0.009
APTT, mean ± SD	29.8 ± 5.4	31.2 ± 6.1	28.3 ± 4.1	0.009
FIB, median (IQR)	1.9 (1.0, 3.2)	2.0 (0.9, 3.3)	1.7 (1.0, 3.0)	0.504
DD, median (IQR)	10.7 (3.5, 32.1)	8.6 (2.7, 20.9)	13.6 (3.8, 36.9)	0.311
ALB, mean ± SD	38.8 ± 5.1	38.8 ± 6.0	38.9 ± 3.8	0.95
Urea, mean ± SD	5.5 ± 2.2	5.3 ± 1.7	5.7 ± 2.7	0.313
Crea, mean ± SD	60.6 ± 25.9	56.9 ± 18.4	64.7 ± 32.1	0.151
LDH, median (IQR)	531.2 (270.4, 1521.0)	287.4 (221.9, 392.2)	1650.0 (871.5, 3013.2)	<0.001
Therapy, *n* (%)				1
Conservative treatments	90 (98.9)	47 (97.9)	43 (100)	
Combined treatments	1 (1.1)	1 (2.1)	0 (0)	
ED30, *n* (%)				0.017
No	35 (38.5)	24 (50)	11 (25.6)	
Yes	56 (61.5)	24 (50)	32 (74.4)	

Abbreviations: ALB, albumin; APL, acute promyelocytic leukemia; APTT, partial thromboplastin time; Crea, creatinine; DD, D-dimer; ED30, 30-day death; EH, epidural hemorrhage; FIB, fibrinogen; HGB, hemoglobin; INR, international normalized ratio; IQR, interquartile range; LDH, lactate dehydrogenase; MSH, multisite hemorrhage; PCH, parenchymal hemorrhage; PLT, platelet; SAH, subarachnoid hemorrhage; SD, standard deviation; SH, subarachnoid hemorrhage; WBC, white blood cell.

Note: Data presented are mean ± SD, median (Q1–Q3), or *N* (%).

Among these patients, 56 (61.5%) experienced mortality within 30 days. Patients in the LDH ≥570 U/L group exhibited higher levels of WBCs, peripheral blasts, INR, and APTT than those in the LDH < 570 U/L group ( *p* < 0.05). The 30-day mortality rate was also higher in the LDH ≥ 570 U/L group than in the LDH < 570 U/L group ( *p* = 0.017). Furthermore, patients in the LDH ≥ 570 U/L group were younger than those in the LDH < 570 U/L group ( *p* = 0.003; Table 1 ).

Table 2 presents the results of the univariate analysis examining the risk factors associated with 30-day mortality in patients with ICH with acute leukemia, reported as hazard ratios (HRs) and their corresponding 95% confidence intervals (CIs). The analysis identified several significant factors associated with 30-day mortality, including the primary disease, bleeding site, WBC count, and log-transformed LDH levels. Conversely, other factors such as sex, age, coinfection, HGB levels, peripheral blasts, PLT count, FIB levels, DD levels, and albumin (ALB) levels were not significantly associated with 30-day mortality ( Table 2 ).

**Table 2:** Univariate analysis of risk factors associated with 30-day mortality in intracranial hemorrhage patients with acute leukemia

Variables	HR (95% CI)	*p* -Value
Sex: female vs. male	1.18 (0.7, 2.01)	0.533
Age	1.01 (1, 1.03)	0.07
Primary disease: non-APL vs. APL	0.49 (0.27, 0.89)	0.02
Bleeding site: ref. = PCH		
SAH or SH or EH	0.37 (0.05, 2.84)	0.34
MSH	2.37 (1.27, 4.44)	0.007
Coinfection: yes vs. no	0.71 (0.41, 1.25)	0.233
WBC	1.0017 (1.0003, 1.0032)	0.015
Peripheral blast	1.0035 (0.9966, 1.0104)	0.324
HGB	0.9952 (0.9834, 1.0072)	0.434
PLT	0.9924 (0.9823, 1.0026)	0.142
FIB	1.05 (0.88, 1.25)	0.603
D-dimer	1.0066 (0.9998, 1.0134)	0.057
ALB	0.96 (0.91, 1.01)	0.104
LDH(log _2_ )	1.23 (1.03, 1.46)	0.02

Abbreviations: ALB, albumin; APL, acute promyelocytic leukemia; CI, confidence interval; DD, D-dimer; EH, epidural hemorrhage; FIB, fibrinogen; HGB, hemoglobin; HR, hazard ratio; LDH, lactate dehydrogenase; MSH, multisite hemorrhage; PCH, parenchymal hemorrhage; PLT, platelet; Ref, reference; SAH, subarachnoid hemorrhage; SH, subarachnoid hemorrhage; WBC, white blood cell.

### Association between Serum Lactate Dehydrogenase Levels and 30-day Mortality Risk in Different Models

Table 3 presents the HRs and their corresponding 95% CIs for the risk of 30-day mortality in relation to serum LDH (Log2) levels and LDH ≥ 570 U/L. The LDH ≥ 570 U/L group exhibited an increased risk of 30-day mortality, with an unadjusted HR of 1.81 (95% CI: 1.07–3.08) compared to the LDH < 570 U/L group. After adjusting for all covariates, the HR and 95% CI remained significant at 2.2 (1.05–4.58). These statistical findings were consistent across all models ( Table 3 ).

**Table 3:** Association between serum lactate dehydrogenase and 30-day mortality risk in different models

Variable	n.total	n.event_%	HR (95 CIs)-Crude model	HR (95 CIs)-Model-I	HR (95 CIs)-Model-II	HR (95 CIs)-Model-III
LDH (log _2_ )	91	56 (61.5)	1.23 (1.03–1.46)	1.33(1.11–1.59)	1.23(1-1.52)	1.27(1.01–1.58)
LDH < 570	48	24 (50)	Ref.	Ref.	Ref.	Ref.
LDH ≥ 570	43	32 (74.4)	1.81 (1.07–3.08)	2.45 (1.37–4.37)	2.05 (1.01–4.15)	2.2 (1.05–4.58)

Abbreviations: ALB, albumin APTT, partial thromboplastin time; CI, confidence interval; Cr, creatinine; FIB, fibrinogen; HGB, hemoglobin; HR, hazard ratio; LDH, lactate dehydrogenase; PLT, platelet; Ref, reference; WBC, white blood cell.

Notes: Crude model adjusted for none. Model-I adjusted for sex, age. Model-II adjusted for sex, age, WBC, PLT, HGB, APTT, FIB. Model-III adjusted for sex, age, WBC, PLT, HGB, APTT, FIB, hypertension, diabetes, trauma, urea, Cr, ALB.

### Subgroup Analyses

**Fig. 2:**
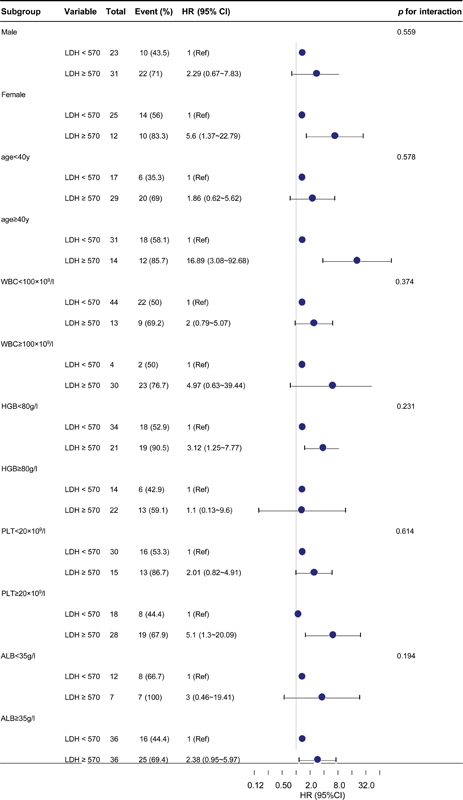
Subgroup analyses of the association between serum LDH levels and 30-day mortality in intracranial hemorrhage patients with acute leukemia. Adjusted for sex, age, WBC, PLT, HGB, APTT, FIB, hypertension, diabetes, trauma, urea, Cr, ALB. ALB, albumin; APTT, partial thromboplastin time; CI, confidence interval; Cr, creatinine; FIB, fibrinogen; HGB, hemoglobin; HR, hazard ratio; LDH, lactate dehydrogenase; PLT, platelet; Ref, reference; WBC, white blood cell.

To investigate whether the association between serum LDH levels and 30-day mortality in patients with ICH with acute leukemia varied across different subgroups, interactive analyses were conducted, stratified based on the results of univariate analysis. The results revealed that no variable exhibited an interactive effect on the association between LDH levels and 30-day mortality ( *p* for interaction > 0.05). However, compared with their respective subgroups, within the LDH ≥ 570 U/L subgroup, individuals aged ≥ 40, with HGB levels < 80 g/L, PLT count < 20 × 10 ^9^ /L, and ALB levels <35 g/L displayed an elevated risk of 30-day mortality. Subgroup analyses were adjusted for covariates, including sex, age, WBC count, PLT, HGB, APTT, FIB, hypertension, diabetes status, trauma, urea, Crea, and ALB ( Fig. 2 ).

## Discussion

Compared with the general population, patients with acute leukemia have a higher likelihood of experiencing ICH, which is a severe complication and a leading cause of death among these individuals. 9 10 11 12 13 In this cohort study, we observed that serum LDH (Log2) levels were associated with an increased risk of 30-day mortality in patients with ICH during the induction phase of acute leukemia. This association remained significant even after adjusting for various factors, including sex, age, primary disease, hypertension, diabetes status, trauma history, bleeding site, coinfection, WBC count, peripheral blast count, HGB levels, PLT count, INR, APTT, FIB, DD levels, urea, Crea, and therapy. More consistent results were observed in the LDH ≥ 570 U/L group than in the LDH < 570 U/L group, and these findings were robust across different levels of adjustment. Previous studies have also suggested that patients with APL and concomitant coagulation abnormalities tend to have a poor prognosis following ICH. 14 15 Therefore, in our study, we categorized the primary disease into two groups, non-APL and APL, as one of the adjusted factors.

In this study, we aimed to characterize the clinical features of ICH in Chinese patients during the induction phase of acute leukemia and identify the prognostic factors associated with 30-day mortality. Previous research has indicated that the 30-day mortality rate for acute leukemia patients with combined ICH is higher than that of patients with other conditions, such as AA and immune thrombocytopenia. 5 In our cohort, the 30-day mortality rate among patients with ICH and acute leukemia was 55.5%. High early mortality remains a significant clinical challenge, and hematologists continue to work on strategies to mitigate these risks. Typically, they assess risk by using laboratory data and the patient's clinical performance status. Previous studies have established associations between high serum LDH levels and increased risk of early mortality in patients with AML. 8 16 17 Elevated LDH levels are often attributed to enhanced glycolytic activity within the tumor and tumor necrosis due to hypoxia, which is linked to a high tumor burden. 8 Most of these studies have independently identified serum LDH as a prognostic factor for AML patients (excluding APL), 3 18 19 with higher LDH levels correlating with shorter survival times. 6 7 However, these studies were conducted in different patient populations, and the relationship between LDH and early mortality was not always considered in conjunction with the patient's vital organ function at admission. 7 20 In our study, we considered several synergistic factors, including ALB, Crea, and blood coagulation parameters, which physicians use to comprehensively assess patients' nutritional status, kidney function, and coagulation function. Furthermore, our study included all patients, regardless of whether they received standard chemotherapy. This approach provides a more objective and comprehensive assessment of whether serum LDH levels impact early mortality in patients with ICH during the induction phase of acute leukemia. Interestingly, our statistical results remained robust in both nonadjusted and adjusted models, consistent with the findings of previous research demonstrating LDH as a prognostic factor in patients with ICH with acute leukemia. 17 18 Notably, the effect was more pronounced in patients with LDH levels ≥ 570 U/L, underscoring the importance of closely monitoring and providing specialized care for these high-risk patients. In high-risk cases, expeditious reduction of the tumor burden may potentially improve the prognosis of this patient group.

This retrospective study had certain limitations. Firstly, a small number of patients may have been excluded due to cases where they initially sought treatment at another hospital and were subsequently transferred to a different facility, resulting in a lack of available imaging data (approximately two or three patients per year). Secondly, being a retrospective study, the data were collected from 2015 to 2020, and for some patients, the date of death was ascertained through telephone follow-up, which may introduce some bias. To mitigate this bias, we conducted thorough interviews with at least two or three family members to accurately determine the patients' survival times. Furthermore, it is worth noting that variations in LDH reagents from different batches could potentially influence test results to some extent. To ensure the reliability of the values, we performed daily internal quality control checks to ensure that the results were within acceptable limits before testing the clinical specimens. Additionally, we participated in an external quality assessment organized by the National Center for Clinical Laboratories annually to verify the accuracy of our test results. Fortunately, our results consistently met the required quality control standards. Moreover, our instruments underwent calibration twice a year as part of routine maintenance procedures. As a result, we can confidently assert the reliability of all testing outcomes.

## Conclusion

Chinese patients with ICH with acute leukemia in the induction phase, who had serum LDH levels ≥ 570 U/L, exhibited a significantly elevated risk of 30-day mortality. Further research is warranted to more conclusively delineate the role of serum LDH in the timely prevention of 30-day mortality in this patient population. This is particularly noteworthy and practical because assessing serum LDH levels is typically a routine and time-efficient test.
